# Impact of Osteoporosis Pharmacotherapy on Functional Outcomes after Ischemic Stroke

**DOI:** 10.3390/jcm12154905

**Published:** 2023-07-26

**Authors:** Jong-Hee Sohn, Chulho Kim, Yerim Kim, So Young Park, Sang-Hwa Lee

**Affiliations:** 1 Department of Neurology, Hallym University Chuncheon Sacred Heart Hospital, Chuncheon 24252, Republic of Korea; deepfoci@hallym.or.kr (J.-H.S.); gumdol52@naver.com (C.K.); 2 Institute of New Frontier Research Team, Hallym University, Chuncheon 24252, Republic of Korea; 3 Department of Neurology, Kangdong Sacred Heart Hospital, Seoul 07441, Republic of Korea; brainyrk@gmail.com; 4 Department of Endocrinology and Metabolism, Kyung Hee University Hospital, Seoul 05278, Republic of Korea; malcoy@hanmail.net

**Keywords:** osteoporosis, osteoporosis pharmacotherapy, bisphosphonate, functional outcomes, ischemic stroke

## Abstract

This study evaluated whether osteoporosis pharmacotherapy (OPT) affected functional outcomes in acute ischemic stroke patients with osteoporosis. Using a single-center registry database, we consecutively registered acute ischemic stroke patients between May 2016 and December 2020. All patients older than 55 years underwent routine bone densitometry within 7 days of stroke onset. OPT prescription was confirmed by reviewing medical records. We classified the patients into OPT and no OPT groups. We performed propensity score matching (PSM) to overcome the imbalance in multiple covariates between the two groups. We investigated whether OPT affected 1-year functional outcomes by multivariate analysis using a PSM cohort. Among 1307 consecutively registered acute ischemic stroke patients, 381 patients were enrolled in this study, of whom 134 (35.2%) were prescribed OPT at discharge, which was maintained for 1 year. In a multivariate analysis using a PSM cohort, the OPT group had a lower risk of dependency (odds ratio [OR], 0.52; 95% confidence interval [CI], 0.27–0.996) and poor functional outcome at 1 year (OR, 0.24; 95% CI, 0.10–0.57). The OPT group also had increased chance of late functional improvement (OR, 6.16; 95% CI, 1.12–33.79). This study showed that OPT could reduce dependency and poor functional outcomes and increase the chance of improving functional outcomes at 3 months and 1 year after ischemic stroke onset, and these findings could be helpful for improving functional outcomes and bone health after ischemic stroke.

## 1. Introduction

The incidence of preexisting osteoporosis was approximately 40% in stroke patients, whereas that of post-stroke osteoporosis was reported to be up to 27% a year after stroke onset [1,2]. Several studies have shown that post-stroke osteoporosis increases the risk of falls and fractures, leading to increased disability and mortality after stroke [3,4,5]. As stroke and osteoporosis are both increasingly prevalent in the aging population, identification and treatment of osteoporosis could be important for improving stroke outcomes. Given the several common risk factors and the causal link between stroke and osteoporosis [6], we hypothesized that osteoporosis treatment could improve outcomes in stroke patients with osteoporosis.

Osteoporosis pharmacotherapy (OPT) is prescribed to 15.5% of stroke patients within 1 year after stroke [7]. However, US guidelines do not have recommendations for testing or treatment for osteoporosis after stroke, and only discuss fall prevention strategies [8]. That is not to say that less importance is being given to osteoporosis; rather, this lack of recommendation could be explained by a scarcity of studies investigating the prognostic significance of osteoporosis as well as limited studies on osteoporosis in stroke patients [9]. A recent study evaluated whether non-pharmacological intervention could improve bone health after stroke [10]. However, no study has investigated the impact of OPT on stroke outcomes after ischemic stroke.

We aimed to investigate whether OPT improves 1-year functional outcomes and to describe the current status of osteoporosis pharmacological strategy in real-world practice.

## 2. Methods

### 2.1. Participants

We consecutively registered acute ischemic stroke patients in a single center between May 2016 and December 2020. All patients older than 55 years underwent routine bone densitometry within 7 days of stroke onset. All female participants in this study were menopausal. Only ischemic stroke patients with osteoporosis were enrolled in this study. Patients who had unstable vital signs or were unconscious (which precludes bone densitometry); those with medical or surgical history that affects bone turnover such as traumatic fracture, spinal surgery, and/or liver, renal, or thyroid disease; those taking medications such as hormonal agents and thiazides; and those with a pre-stroke modified Rankin Scale (mRS) score > 2 were excluded.

### 2.2. Data Collection and Definition of Parameters

The following data were obtained directly from the registry database: demographic characteristics (age and sex); stroke risk factors and medical history: previous stroke, hypertension, diabetes mellitus, hyperlipidemia, atrial fibrillation, current smoking status, and prior antithrombotic drug use; clinical characteristics and acute stroke treatment, including the initial National Institutes of Health Stroke Scale (NIHSS) score, ischemic stroke mechanism according to the TOAST criteria with some modifications, body mass index, and systolic blood pressure; laboratory data, including osteocalcin, 25 (OH) vitamin D, C-terminal telopeptide of type 1 collagen, calcium, phosphorus, white blood cell count, hemoglobin, creatinine, initial random glucose, low-density lipoprotein, and glycated hemoglobin levels; and functional status defined by the mRS scores at 3 months and 1 year.

There was no specific protocol for prescribing osteoporosis treatment, and prescribing medications was entirely up to the physicians. Therefore, whether patients were prescribed OPT at discharge was confirmed by a retrospective review of their electronic medical records. We entered the ingredients and usage of each drug into the study database. Drug compliance was determined according to whether patients were prescribed drugs regularly without follow-up loss during 1 year after stroke onset. In addition, we determined the presence of fractures between 3 months and 1 year of stroke onset via telephone interview by a trained coordinator and reviewed the patients’ medical records at 1 year after stroke onset.

The primary outcome measures were proportion of dependency (mRS > 2) and poor functional outcomes (mRS > 3) at 1 year (mRS scores: 0 = no symptoms; 1 = no significant disability; 2 = slight disability; 3 = moderate disability, requiring some help but able to walk without assistance; 4 = moderately severe disability, unable to attend to own bodily needs without assistance; 5 = severe disability, requiring constant nursing care and attention, bedridden; 6 = death) [11]. The secondary outcome measure was proportion of late functional improvement defined as an mRS decrease ≥ 1 point between 3 months and 1 year [12,13]. To identify the current status of OPT in our institution, we retrospectively reviewed the physicians’ reasons for not prescribing OPT medications at discharge and which OPT drugs were prescribed by physicians to stroke patients.

### 2.3. Bone Mineral Density Measurements

The bone mineral densities (BMD) at the lumbar spine and femoral neck were estimated using dual-energy X-ray absorptiometry (Horizon W DXA system; Hologic, Marlborough, MA, USA). The coefficient of variation was approximately 1% for the BMD of the lumbar spine and femoral neck. The lumbar spine BMD was measured as the mean BMD from the first to the fourth lumbar vertebra. The femoral neck BMD was measured on only the right side. Osteoporosis was defined as a BMD at the lumbar spine or femoral neck of at least 2.5 standard deviations (SDs) below the reference mean (T score < −2.5) [14].

### 2.4. Statistical Analysis

We hypothesized that OPT could be associated with 1-year functional outcomes. We classified patients into OPT and no OPT groups. We compared geographical, clinical, and laboratory variables using Pearson’s chi-square test or Fisher’s exact test for categorical variables and the Student *t*-test or Mann–Whitney U test for continuous variables, as appropriate.

To overcome the possible imbalance of covariates and confounding factors between the OPT and no OPT groups, we performed propensity score matching (PSM). The propensity score for each group was defined as the probability of the presence of OPT, given the patients’ initial demographics, vascular risk factors, and laboratory values in the baseline logistic regression analysis. Based on these propensity scores, the OPT and no OPT groups were matched 1:1 using the nearest neighbor method (PSM cohort). The multivariate analysis was adjusted for variables selected based on *p* < 0.1 in the comparisons of groups stratified according to OPT, and all clinically plausible associations with each outcome variable were determined. All data analyses were performed with IBM SPSS version 21.0 software (IBM Corporation, Armonk, NY, USA) and moonBook and MatchIt of R version 4.0.3 (R Core Team 2020, R Foundation for Statistical Computing, Vienna, Austria).

As a sensitivity analysis, we evaluated the independent effects of OPT on 1-year functional outcome measures and performed a binary logistic regression analysis using the total cohort. Crude and adjusted odds ratios (ORs) and 95% confidence intervals (CIs) were calculated.

## 3. Results

**Figure 1:**
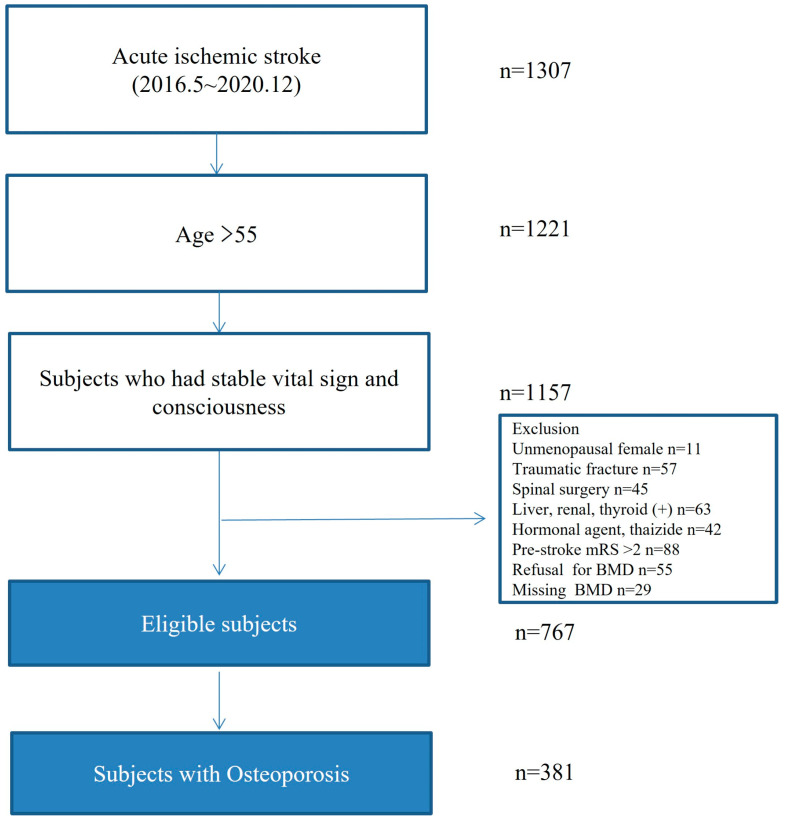
Flow chart of this study.

Among 1307 consecutively registered acute ischemic stroke patients, 767 met the inclusion criteria during the study period. Of these 767 patients, 381 (49.7%) had osteoporosis. A total of 134 (35.2%) were prescribed OPT at discharge, which was maintained for 1 year (Figure 1). The OPT group had more female patients and lower BMI and stroke severity than the no OPT group. The vertebral and femur fracture rates did not differ between the OPT and no OPT groups (Table 1). Meanwhile, most patients with osteoporosis were not prescribed OPT because of physician malpractice and mistake during hospitalization (81.4%, 201/247). The other reasons were patients’ refusal (*n* = 24), stopping of medication due to adverse effects (*n* = 3), and unknown reasons (*n* = 20). It was confirmed that the majority of the OPT group (91.0%, 122/134) received prescriptions after consulting with an endocrinologist. In the OPT group, intravenous zoledronate once a year was the preferred medication during hospitalization (59.0%, 79/134), followed by oral risedronate once a week (*n* = 34), oral ibandronate once a month (*n* = 15), and others (*n* = 6).

After propensity score matching (PSM), 134 patients who were on OPT were matched 1:1 with patients who were not on OPT. In the PSM cohort, the baseline characteristics of the two groups were similar and reasonably balanced (Table 1).

**Figure 2:**
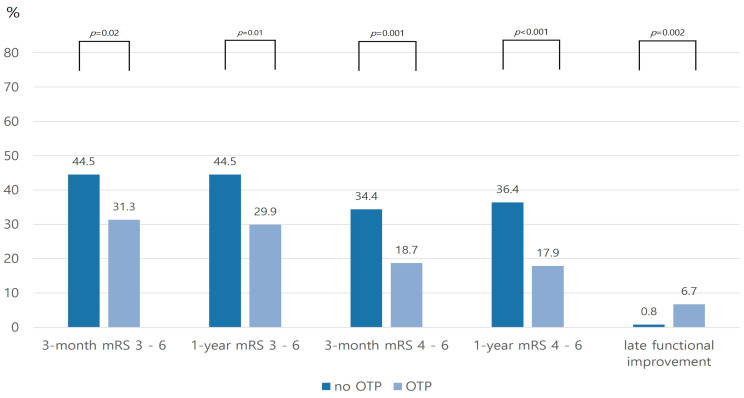
Distribution of stroke outcomes according to OPT.

**Figure 3:**
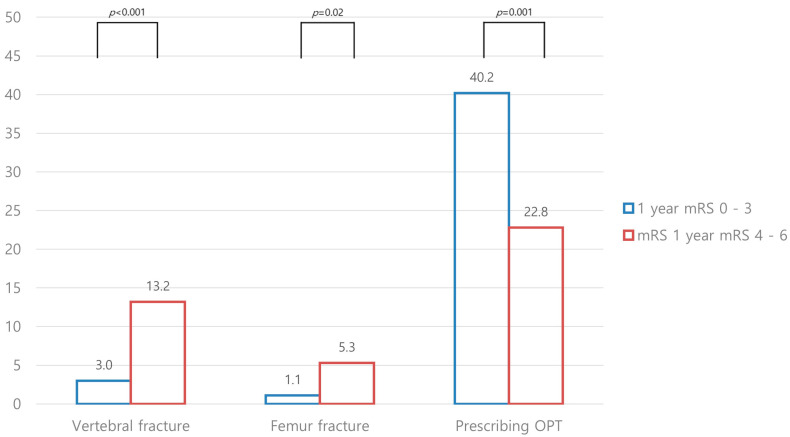
Distribution of vertebral and femur fractures according to mRS score.

With respect to primary outcome measures, the proportions of dependency and poor functional outcome at 1 year were significantly lower in the OPT group than in the no OPT group (dependency, 29.9% versus 44.5%, *p* = 0.01; poor functional outcome, 17.9% versus 36.4%, *p* < 0.001). These outcomes were also lower in the OPT group than in the no OPT group at 3 months (Figure 2). The secondary outcome measure, late functional improvement, was higher in the OPT group than in the no OPT group (6.7% versus 0.8%, *p* = 0.002). In addition, the vertebral and femur fracture rates at 1 year after stroke onset were higher in patients with dependency than in independent patients (vertebral fracture, 13.2% versus 3.0%, *p* < 0.001; femur fracture, 5.3% versus 1.1%, *p* = 0.02). In contrast, the OPT prescription rate was lower in the poor outcome group (40.2% versus 22.7%, *p* = 0.001; Figure 3).

The multivariate logistic regression analysis using PSM cohort revealed that OPT could reduce the risk of dependency (OR [95% CI]: 0.52 [0.27–0.996]) and poor functional outcome at 1 year (OR [95% CI]: 0.24 [0.10–0.57]) (Table 2). As a sensitivity analysis, multivariate analysis using the total cohort also showed that OPT was associated with the reducing the risk of dependency and poor functional outcomes at 1 year (Supplementary Table S1). In addition, OPT potentially reduced the poor functional outcomes at 3 months in multivariate analysis using both total and PSM cohort (Table 3). In addition, multivariate analysis indicated that OPT significantly increased the late functional improvement (OR [95% CI]: 6.16 [1.12–33.79]). The multivariate analysis revealed that any fractures also increased the dependency and poor functional outcome at 1 year.

## 4. Discussion

The present study hypothesized that OPT affects functional outcome in acute ischemic stroke patients with osteoporosis, and the main findings were the following: (1) the OPT group had greater dependency and poorer functional outcome at 1 year of stroke onset; (2) the OPT group had more late functional improvement between 3 months and 1 year; and (3) the multivariate analysis showed that OPT was associated with a reduced risk of dependency and poor functional outcome at 1 year and an increased chance of late functional improvement. 

A recent registry-based study revealed that 15.5% of overall stroke patients were prescribed osteoporosis medication for prevention of fracture within 1 year after stroke [7]. Although the prescription rate of OPT (35.2%) in our single-center registry was higher than that of the above-mentioned study, a substantial gap between clinician’s awareness and the importance of OPT after ischemic stroke remains in real-world practice. Although this is a single-center study, clinicians’ indifference could contribute to the low prescription rate of OPT after ischemic stroke. This could be explained by the fact that current guidelines have no clear recommendations for prevention of osteoporosis after stroke due to lack of evidence on this issue.

A few clinical trials have investigated the impact of anti-osteoporosis medications on post-stroke osteoporosis and prevention of fracture after stroke [15,16]. Meanwhile, in this study, we showed that OPT could be associated with improved functional outcomes. The occurrence of fracture was not different according to OPT in the bivariate analysis, and OPT was not associated with improved fracture in the multivariate analysis of our database (OR [95% CI]: 1.30 [0.58–2.99], *p* = 0.54). In addition, fracture dependently potentially reduced the 1-year dependency and poor functional outcomes. Hence, we inferred that OPT, as well as physiotherapy, could be associated with improved long-term functional outcomes, regardless of fracture improvement, which is a novel finding. However, we could not determine the causal link between OPT and improved functional outcome. Previous in vitro studies showed that bisphosphonate has a neuroprotective effect on neurological disorders via the mevalonate pathway through inhibition of protein prenylation; amelioration of glutamic-induced toxicity, cell apoptosis, and calcium overloading; and attenuation of neurotoxic substances, such as pro-inflammatory cytokines and nitric oxide [17,18,19,20,21,22]. Hence, we carefully speculated that OPT could improve functional status after ischemic stroke through the aforementioned neuroprotective mechanisms. Further in vivo studies and large clinical trials are warranted to demonstrate this issue.

Both vertebral and femur fractures between 3 months and 1 year of stroke onset had higher rates in patients with poor functional outcomes in this study. However, the prescription rate of OPT was lower in those patients. Presumably, the adherence and prescription of OPT tended to be better for patients with better functional status as was shown in a previous observational study [23]. Based on similar results, it can be inferred that, in our study, the patient’s neurological symptoms were poor, making it less likely for OPT to be prioritized. However, considering that reduced mobility and decreased bone load due to paresis are key mechanisms of post-stroke osteoporosis, a vicious cycle could occur, leading to further deterioration of the patient’s functional status if OPT is not administered [6]. Since the present study demonstrated that OPT can improve late functional status, we cautiously suggest that patients with poorer functional status may benefit from OPT. While our results were based on a single-center experience and could not be generalized, early initiation of OPT with physiotherapy after stroke could be considered.

The adequate management of post-stroke osteoporosis is not elucidated in the current guidelines. Management of post-stroke osteoporosis should be considered with beneficial and adverse effects as well as comprehensive risk assessment. Previous studies suggested that vitamin D supplements may be beneficial to osteoporosis prevention in stroke patients because vitamin D deficiency commonly occurs after stroke [9,24]. Although several studies evaluated the impact of oral bisphosphonate on fracture prevention after stroke [6], it was not generally prescribed for the stroke patients in the present study due to the following reasons: administration of oral bisphosphonate is limited in patients with dysphagia and those who could not remain upright [9,25]; oral bisphosphonate has gastrointestinal adverse effects such as difficulty in swallowing, gastric inflammation, and ulcer [26]; limited ability of patients to take pills routinely for weeks or months in those who had cognitive impairment due to aging or stroke complication. Hence, despite the risk of cardiovascular adverse effects [27] and hypocalcemia [28,29], annual administration of intravenous zoledronate is favored because its benefits offset the several adverse effects of oral bisphosphonate administration in stroke patients in our database [7]. Based on this evidence, vitamin D and calcium supplement could be recommended, and intravenous zoledronate is also a reasonable medication for stroke patients [25]. Since evidence on this issue remains sparse, the OPT strategy for post-stroke osteoporosis should be investigated with large-scale clinical trials.

This study has some limitations. First, this retrospective study was based on a single-center registry database, making it difficult to investigate the causal relationship between OPT and improvement in functional outcomes after stroke. Hence, we caution against generalizing our results. Nonetheless, we consecutively and consistently collected BMD and bone turnover markers in acute ischemic stroke patients for the study period. Second, the baseline functional status at discharge could be poor, despite being statistically insignificant between the no OPT and OPT groups (mRS at discharge > 2: 39.3% and 31.2%, respectively, *p* = 0.15). Therefore, it is possible that the physicians were already biased in prescribing OPT. However, we analyzed whether OPT affects the secondary outcome measure, late functional improvement, which predicts continued recovery and delayed improvement. Third, the determination of the presence and site of fracture was based on telephone interview of patients, or their relatives and a review of electrical medical records and imaging indicated fractures. Due to the retrospective nature of this data collection method, the accuracy of the fracture sites may be uncertain, and therefore, the reliability of the data may be limited. However, it is important to note the impact of OPT on functional improvement after ischemic stroke, rather than specifically focusing on fractures. Regarding our results, we have shown the actual treatment status of osteoporosis after stroke and the treatment outcomes in real-world practice. Fourth, recent OPTs, such as IgG2 monoclonal antibody (denosumab) and anabolic agents (teriparatide, abaloparatide, and romosozumab), were not available in our study, and we were unable to determine the reason why those OPTs were not available in our registry. Since the progression of bone loss after stroke could be accelerated in the first 6 to 12 months after stroke onset, denosumab could be given every 6 months to prevent osteoporosis after ischemic stroke. Fifth, although we excluded patients taking thiazide, other medications that affect bone turnover, such as statins, anticoagulants (warfarin and heparin), and proton pump inhibitors, were not excluded in this study [30,31,32].

In real-world practice, clinicians are generally not concerned with treating osteoporosis after stroke due to the lack of adequate recommendation in guidelines. However, this study showed that OPT could reduce dependency and poor functional outcome and increase the chance of improving functional outcome at 3 months and 1 year after ischemic stroke onset. Thus, although our study was based on a single-center experience, our main results suggest the importance of screening for osteoporosis and OPT in patients with ischemic stroke, thereby improving both stroke outcomes and bone health after ischemic stroke.

## Figures and Tables

**Table 1:** Baseline characteristics according to osteoporosis pharmacotherapy (OPT) in the total cohort and the PSM cohort.

	Total Cohort			PSM Cohort		
	No OPT (*n* = 247)	OPT (*n* = 134)	*p*-Value	No OPT (*n* = 134)	OPT (*n* = 134)	*p*-Value
Age, years (SD)	75.5 (11.0)	75.8 (10.9)	0.73 ^†^	76.0 (10.2)	75.8 (10.9)	0.89
Female, *n* (%)	171 (69.2)	106 (79.1)	0.04 *	106 (79.1)	106 (79.1)	1.000
BMI, kg/m^2^ (IQR)	23.0 (21–25)	22.2 (20.0–25.0)	0.03 ^‡^	22.6 (20.8–24.0)	22.2 (20.0–25.0)	0.91
Initial NIHSS score (IQR)	4 (1–8)	3 (1–5)	0.01 ^‡^	2 (1–5)	3 (1–5)	0.53
Stroke subtypes, *n* (%)			0.32 *			0.75
SVO	72 (29.1)	42 (31.3)		48 (35.8)	42 (31.3)	
LAA	72 (29.1)	47 (35.1)		39 (29.1)	47 (35.1)	
CE	55 (22.3)	20 (14.9)		20 (14.9)	20 (14.9)	
Others	48 (19.4)	25 (18.7)		27 (20.1)	25 (18.7)	
Prior stroke, *n* (%)	58 (23.5)	38 (28.4)	0.32 *	36 (26.9)	38 (28.4)	0.89
Hypertension, *n* (%)	156 (63.2)	88 (65.7)	0.66 *	94 (70.1)	88 (65.7)	0.51
Diabetes mellitus, *n* (%)	82 (33.2)	36 (26.9)	0.21 *	45 (33.6)	36 (26.9)	0.29
Hyperlipidemia, *n* (%)	32 (13.0)	16 (11.9)	0.87 *	5 (11.2)	16 (11.9)	1.00
Current smoking, *n* (%)	16 (6.5)	6 (4.5)	0.50 *	6 (4.5)	6 (4.5)	1.00
Prior antiplatelet, *n* (%)	69 (27.9)	47 (35.1)	0.16 *	48 (35.8)	47 (35.1)	1.00
Prior anticoagulation, *n* (%)	23 (9.3)	7 (5.2)	0.17 *	6 (4.5)	7 (5.2)	1.00
Prior statin, *n* (%)	25 (10.1)	20 (14.9)	0.19 *	18 (13.4)	20 (14.9)	0.86
Reperfusion therapy, *n* (%)			0.27 *			0.37
No	209 (84.6)	120 (89.6)		125 (93.3)	120 (89.6)	
IVT	15 (6.1)	9 (6.7)		8 (6.0)	9 (6.7)	
EVT	10 (4.0)	2 (1.5)		0 (0.0)	2 (1.5)	
IVT+EVT	13 (5.3)	3 (2.2)		1 (0.7)	3 (2.2)	
Lesions, *n* (%)			0.71 *			0.95
Anterior	198 (80.2)	103 (76.9)		101 (75.4)	103 (76.9)	
Posterior	35 (14.6)	24 (17.9)		26 (19.4)	24 (17.9)	
Multiple	13 (5.3)	7 (5.2)		7 (5.2)	7 (5.2)	
Rehabilitation, *n* (%)	57 (23.1)	35 (26.1)	0.53 *	36 (26.9)	35 (26.1)	1.00
Vertebral fracture, *n* (%)	12 (4.9)	11 (8.2)	0.26 *	9 (6.7)	11 (8.2)	0.82
Femur fracture, *n* (%)	6 (2.4)	3 (2.2)	0.91 *	4 (3.0)	3 (2.2)	1.00
mRS at discharge > 2, *n* (%)	92 (38.0)	42 (30.2)	0.15 *	42 (31.3)	42 (31.3)	1.00
Laboratory findings						
Osteocalcin, ng/mL (SD)	13.4 (16.9)	14.2 (12.5)	0.59 ^†^	13.4 (16.6)	(14.2 ± 12.5)	0.64
CTx, ng/mL (SD)	0.5 (1.2)	0.5 (0.5)	0.94 ^†^	0.6 (1.7)	0.5 (0.5)	0.60
25 (OH) Vitamin D, ng/mL (SD)	16.1 (8.6)	16.6 (10.4)	0.63 ^†^	16.2 (8.3)	16.6 (10.4)	0.78
Calcium, mg/dL (SD)	9.1 (0.4)	9.1 (0.5)	0.31 ^†^	9.1 (0.4)	9.1 (0.5)	0.51
Phosphorus, mg/dL (SD)	3.3 (0.5)	3.4 (0.7)	0.11 ^†^	3.3 (0.5)	3.4 (0.7)	0.17
Creatinine, mg/dL (SD)	1.0 (0.9)	1.0 (0.9)	0.93 ^†^	1.0 (0.9)	1.0 (0.9)	0.81
Hemoglobin, mg/dL (SD)	13.0 (2.2)	12.9 (1.9)	0.52 ^†^	12.9 (2.0)	12.9 (1.9)	0.87
Platelet count, uL/10^3^ (SD)	223.6 (66.9)	228.3 (58.8)	0.50 ^†^	230.4 (58.0)	228.3 (58.8)	0.77
LDL, mg/dL (SD)	97.8 (38.3)	99.2 (33.3)	0.72 ^†^	98.9 (38.5)	99.2 (33.3)	0.96
HbA1c, % (SD)	6.0 (1.3)	5.9 (0.9)	0.15 ^†^	5.9 (1.2)	5.9 (0.9)	0.60
CRP, mg/dL (SD)	11.7 (29.7)	8.6 (30.1)	0.34 ^†^	8.9 (19.9)	8.6 (30.1)	0.93
Initial glucose, mg/dL (SD)	135.4 (56.5)	131.9 (39.5)	0.49 ^†^	136.8 (62.9)	131.9 (39.5)	0.45
SBP, mmHg (SD)	149.1 (28.1)	149.0 (26.7)	0.97 ^†^	146.8 (28.9)	149.0 (26.7)	0.51
T3, μU/mL (SD)	73.2 (23.4)	74.6 (21.5)	0.57 ^†^	75.6 (22.1)	74.6 (21.5)	0.73
fT4, μU/mL (SD)	1.0 (0.18)	1.0 (0.2)	0.49 ^†^	1.0 (0.2)	1.0 (0.2)	0.91
TSH, μU/mL (SD)	1.6 (1.2)	1.6 (1.2)	0.91 ^†^	1.5 (1.0)	1.6 (1.2)	0.60

Abbreviations: OPT, osteoporosis pharmacotherapy; SD, standard deviation; IQR; interquartile range; BMI, body mass index; SVO, small vessel occlusion; LAA, large artery atherosclerosis; CE; cardioembolism; IVT, intravenous thrombolysis; EVT, endovascular treatment; CTx, c-terminal telopeptide; LDL, low density lipoprotein; HbA1c, glycated hemoglobin; CRP, C reactive protein; SBP, systemic blood pressure; fT4, free T4; TSH, thyroxine stimulation hormone. * Calculated using the chi-square test. ^†^ Calculated using Student’s *t*-test. ^‡^ Calculated using the Mann–Whitney U test.

**Table 2:** Multivariate analysis shows impact of OPT on 1-year stroke outcomes using PSM cohort.

	1-Year Dependency (mRS 3–6)		1-Year Poor Functional Outcome (mRS 4–6)		Late Improvement Functional Outcome	
	OR	95% CI	OR	95% CI	OR	95% CI
OPT	0.52	0.27–0.996	0.24	0.10–0.57	6.16	1.12–33.79
Age	1.05	1.01–1.09	1.05	1.001–1.09	1.08	0.99–1.17
Female	0.996	0.42–2.37	0.62	0.22–1.72	0.90	0.17–4.95
BMI	0.92	0.82–1.03	0.97	0.85–1.11	0.91	0.73–1.14
Initial NIHSS	1.32	1.19–1.45	1.42	1.26–1.59	0.99	0.82–1.18
Stroke subtypes						
SVO	ref		ref		ref	
LAA	1.39	0.63–3.08	1.21	0.44–3.29	0.90	0.17–4.63
CE	1.17	0.40–3.38	1.02	0.28–3.68	0.73	0.09–6.08
Others	0.88	0.33–2.31	1.04	0.33–3.30	0.21	0.02–3.00
Rehab	1.35	0.68–2.70	0.29	0.11–0.77	0.75	0.16–3.43
Fracture	10.57	3.47–32.21	15.95	4.87–52.26	0.27	0.02–3.68
Vitamin D	0.98	0.94–1.01	0.97	0.93–1.02	0.93	0.85–1.02
Calcium	1.43	0.71–2.92	1.62	0.68–3.89	0.30	0.08–1.11
Phosphorus	1.17	0.72–1.91	1.30	0.67–3.89	0.92	0.32–2.68
Osteocalcin	1.02	0.99–1.04	1.01	0.97–1.05	0.95	0.88–1.03
CTx	0.91	0.63–1.39	0.50	0.10–2.51	1.03	0.50–2.15

Abbreviations: OPT, osteoporosis pharmacotherapy; PSM, propensity score matching; BMI, body mass index; NIHSS, National Institutes of Health Stroke Scale; SVO, small vessel occlusion; LAA, large artery atherosclerosis; CE, cardioembolism; Rehab, rehabilitation; CTx, C-terminal telopeptide.

**Table 3:** Multivariate analysis shows impact of OPT on 3-month stroke outcomes.

	3-Month Poor Functional Outcome in PSM Cohort (mRS 4–6)		3-Month Poor Functional Outcome in Total Cohort (mRS 4–6)	
	OR	95% CI	OR	95% CI
OPT	0.30	0.13–0.72	0.49	0.25–0.94
Age	1.05	1.01–1.10	1.03	1.00–1.06
Female	0.45	0.16–1.27	0.96	0.48–1.92
BMI	0.95	0.83–1.09	0.95	0.48–1.92
Initial NIHSS	1.43	1.26–1.61	1.29	1.20–1.39
Stroke subtypes				
SVO	ref		ref	
LAA	1.11	0.39–3.17	1.13	0.50–2.58
CE	1.79	0.51–6.29	1.83	0.71–4.69
Others	1.22	0.37–4.00	1.82	0.75–4.45
Rehab	0.16	0.05–0.49	0.09	0.03–0.24
Vitamin D	0.96	0.91–1.01	0.97	0.94–1.01
Calcium	1.24	0.51–3.03	0.67	0.32–1.39
Phosphorus	1.41	0.77–2.59	1.11	0.63–1.94
Osteocalcin	1.01	0.97–1.05	0.997	0.97–1.02
CTx	0.44	0.09–2.21	0.77	0.25–2.39

Abbreviations: OPT, osteoporosis pharmacotherapy; PSM, propensity score matching; mRS, modified Rankin Scale; BMI, body mass index; NIHSS, National Institutes of Health Stroke Scale; SVO, small vessel occlusion; LAA, large artery atherosclerosis; CE, cardioembolism; Rehab, rehabilitation; CTx, C-terminal telopeptide.

## Data Availability

All datasets generated and/or analyzed during the current study are not publicly available as use of the data requires ethical approval. To inquire about access to the study data, contact the corresponding author.

## Supplementary Materials

The following supporting information can be downloaded at: https://www.mdpi.com/article/10.3390/jcm12154905/s1, Table S1: Multivariate analysis showing the impact of OPT on 1-year stroke outcomes for the total cohort.
