# Supplementary material for: Impact of Osteoporosis Pharmacotherapy on Functional Outcomes after Ischemic Stroke
Source: J Clin Med. 2023 Jul 26;12(15):4905. doi: 10.3390/jcm12154905 (PMC10420261; doi:10.3390/jcm12154905)
Supplement: Supplementary file 1 [file jcm-12-04905-s001.zip › jcm-2429293-supplementary.pdf]

Supplementary Table S1. Multivariate analysis showing the impact of OPT on 1-year stroke outcomes for the total cohort.

	1-year dependency (mRS 3-6)		1-year poor functional outcome (mRS 4-6)		Late improvement functional outcome	
	OR	95% CI	OR	95% CI	OR	95% CI
OPT	0.53	0.30-0.97	0.32	0.16-0.65	8.11	1.56-42.18
Age	1.04	1.01-1.07	1.02	0.99-1.05	1.07	0.99-1.15
Female	0.94	0.49-1.78	0.95	0.45-1.99	1.06	0.20-5.73
BMI	0.96	0.88-1.05	0.98	0.89-1.07	0.92	0.74-1.16
Initial NIHSS	1.27	1.19-1.37	1.28	1.19-1.38	0.97	0.80-1.16
Stroke subtypes						
SVO	ref		ref		ref	
LAA	1.58	0.80-3.13	1.36	0.59-3.14	0.93	0.18-4.79
CE	1.39	0.57-3.38	1.37	0.50-3.75	0.68	0.07-6.40
Others	1.39	0.63-3.10	1.67	0.65-4.28	0.25	0.02-3.83
Rehab	1.18	0.65-2.16	0.19	0.08-0.45	0.74	0.16-3.58

Fracture	9.17	3.45-24.40	12.36	4.55-33.62	0.41	0.03-5.44
Vitamin D	0.98	0.95-1.01	0.98	0.95-1.01	0.94	0.86-1.03
Calcium	0.98	0.52-1.85	1.08	0.52-2.24	0.27	0.07-1.05
Phosphorus	0.94	0.60-1.47	0.97	0.55-1.72	0.86	0.31-2.39
HbA1c	0.72	0.51-1.02	0.51	0.33-0.78	1.38	0.73-2.60
Initial glucose	1.01	1.001-1.02	1.02	1.01-1.03	0.995	0.98-1.02
T3	0.994	0.98-1.01	0.99	0.98-1.01	1.01	0.98-1.05
